# Evaluation of Hydro-Alcoholic Extract of *Trifolium Pratens L.* for Its Anti-Cancer Potential on U87MG Cell Line

**DOI:** 10.22074/cellj.2018.5380

**Published:** 2018-05-28

**Authors:** Mozafar Khazaei, Mona Pazhouhi, Saber Khazaei

**Affiliations:** 1 Fertility and Infertility Research Center, Kermanshah University of Medical Sciences, Kermanshah, Iran; 2 Department of Endodontics, Dental Research Center, Isfahan University of Medical Sciences, Isfahan, Iran

**Keywords:** Apoptosis, Autophagy, Glioblastoma Multiforme, Temozolomide

## Abstract

**Objective:**

Glioblastoma multiforme is the most malignant form of brain tumors. *Trifolium pratense L.* has been suggested for cancer treatment in traditional medicine. Here we have investigated the effects of T. pratense extract on glioblastoma multiforme cell line (U87MG).

**Materials and Methods:**

In this experimental study the effect of *T. pratense* extract on cell viability was investigated using trypan blue staining, MTT assay, and lactate dehydrogenase activity measurement. Apoptosis and autophagy cell death were detected by fluorescent staining. Nitric oxide (No) production was measured using Griess reaction. Expression levels of some apoptotic and autophagic-related genes were detected using real-time polymerase chain reaction (PCR). The combination effects of *T. pratense* extract and temozolomide (TMZ) were evaluated by calculating the combination index and dose reduction index values.

**Results:**

After treatment with *T. pratense* extract, the cell viability was significantly reduced in a time- and dose- dependent manner (P<0.05). Apoptosis and autophagy of U87MG cells were significantly increased (P<0.05). Also, T. pratense extract significantly decreased NO production (P<0.05) by U87MG cells. Combination of TMZ and T. pratense extract had a synergistic cytotoxic effect.

**Conclusion:**

T. pratense showed anti-cancer properties via induction of apoptosis and autophagy cell death.

## Introduction

Glioblastoma multiforme (GBM) is the most aggressive and most common type of the malignant astrocytic brain tumors. Surgical resection (which is usually incomplete because of the proximity of the tumor to vital brain structures), radiotherapy, and chemotherapy are the currently used conventional treatments (1). DNA alkylating agents are among the oldest class of anti-cancer drugs still commonly used, which play an important role in the different types of tumor treatments (2). 

Temozolomide (TMZ), an imidazole derivative, is an oral chemotherapy agent commonly used to control the growth of GBM tumors. Due to its lipophilic properties, it readily passes the blood brain barrier and spontaneously hydrolyzes under physiological conditions to its active form. It can methylate DNA at the O6 and N7 positions of guanine. These methylated bases disturb DNA replication and cell cycle, therefore activating apoptosis death pathway. However, GBM are among the most resistant tumors to chemotherapy treatment, because of cell DNA repair system. 

The most important mechanism of TMZ resistance is the DNA repair enzyme O6-methylguanine methyltransferase (MGMT), which removes the cytotoxic O6-methylguanine and counteracts the effect of TMZ. GBM patients survive, on average, between 12 and 15 months, despite conventional therapy (3). So it seems necessary to identify new strategies to treat this kind of cancer. Currently, many attempts have been made to overcome drug resistance, using combination therapy with multiple anti-cancer agents. Different anticancer agents affect different targets and cell subpopulations and therefore can enhance the therapeutic effects, reduce dose and side effects and prevent or delay the induction of drug resistance. Recent studies have shown that combination of TMZ with some herbal agents enhances its effectiveness on glioblastoma cells (4). 

For over 40 years, natural products, in either unmodified or synthetically modified forms, have played an important role in cancer therapy. In fact, over 60% of currently used chemotherapy drugs have been isolated from natural products, mostly of plant origin (5). In the 1950s the potential of using natural products as anti-cancer drugs was confirmed by U.S National Cancer Institute (NCI), and from that time there is a growing interest in discovery of naturally occurring anticancer drugs. Some of such drugs that are used against cancer include vinca alkaloids (vincristine, vinblastine, vindesine, vinorelbine), taxanes (paclitaxel, docetaxel), podophyllotoxin and its derivatives (etoposide, teniposide), camptothecin and its derivatives (topothecan, irinothecan), anthracyclines (doxorubicin, daunorubicin, epirubicin, idarubicin) and others (6).

According to other studies the mechanisms of plants for anti-cancer properties are numerous and most of them cause apoptotic cell death induction via intrinsic or extrinsic mechanisms, and CASPASE- and/or *P53*-dependent or independent pathways. Also, anti-cancer potentials of some plants are through induction of autophagy, necrosis-like programmed cell death, mitotic catastrophe, and senescence (7).

*Trifolium pratense L.*, a member of *Leguminosae* or *Fabaceae family*, is a short-lived biennial plant, which has been used as a fodder crop for its nitrogen fixation potential, increases soil fertility and is considered as a health food for humans. It is probably native to Europe, Western Asia, and northwest Africa, but it has been naturalized in other continents (8). Many isoflavones extracted from T. pratense are available nowadays as dietary supplements (9). This plant has also been suggested in traditional medicine as a treatment for some human diseases such as whooping cough, asthma, eczema and certain eye diseases (10). A study documented the chemical profile of *Trifolium pratense L.* extract using the high-performance liquid chromatography-ultraviolet (HPLC-UV) chromatogram. The results showed that *Trifolium pratense L.* extract was composed of isoflavones, flavonoids, pterocarpans, coumarins and tyramine (11). Its main isoflavones are biohanin A, formononetin, daizdein, genistein, pratensein, prunetin, pseudobaptigenin, calycosin, methylorobol, afrormosin, texasin, irilin B and irilone (12).

Despite current remarkable progress in cancer therapeutics, it remains the leading cause of death in the world. So the discovery and development of new therapeutic strategies seems to be necessary. Although *Trifolium pratense L.* has been suggested for cancer treatment in traditional medicine, but there are currently no literature reports about anti-cancer potentials of this plant. Therefore, the present study was performed to determine the effects of *T. pratense* hydroalcoholic extract on a glioblastoma cell line.

## Materials and Methods

### Cell line and reagents

For this *in vitro* experimental study, human GBM cell line (U87MG) was obtained from the National Cell Bank of Iran (NCBI). TMZ, trypsin, 3-(4, 5-dimethylthiazol2- yl)-2, 5-diphenyltetrazolium bromide (MTT), acridin orange (AO), ethydium bromide (EB) and propidium iodide (PI) were purchased from Sigma-Aldrich Chemical Co (St. Louis, MO, USA). Dulbecco’s modified eagle medium/Ham’s F12 nutrient mixture (DMEM/ F12) and fetal bovine serum (FBS) were purchased from Gibco (Gaithersburg, MD, USA). All experiments were performed in triplicates and were repeated independently at least three times. The study was approved by Ethical Committee of Kermanshah University of Medical Sciences, Kermanshah, Iran (Code: kums.res.1395.46).

### Preparation of crude extracts

*T. pratense* seeds were cultured in spring of 2017 in a farm and identified in terms of species by a botanist (Kermanshah University of medical sciences, Kermanshah, Iran). Aerial parts of the plants were dried and powdered, and 15g of the powder were dissolved in 150 mLof 70% ethanol for 48 hours in the dark. Then it was filtered through filter paper (Watman, grade 42) and dried to allow for evaporation of the alcohol at room temperature. Finally, the powder was dissolved in a serum-free cell culture medium, and passed through a 0.22 µm filter (13).

### Cell culture and treatment

U87MG cell line was grown in cell culture flasks containingDMEM/F12 supplemented with 10% FBS and no antibiotics. Cells were maintained at 37.C in a humidified chamber containing 5% CO_2_ (14). TMZ were dissolved in DMSO at astock concentration of 100 mM and stored at -20.C until use. The cell line was treated with *T. pratense* extract (6.25, 12.5, 25, 50, 100, 200 and 400 µg/mL).

### Trypan blue dye exclusion

U87MG cells were seeded in 24-well plates at 7×10^4^ cells per well and incubated overnight. Then, the cell culture medium was replaced with fresh serum-free medium containing various concentrations of T. pratenseextract. The cells were incubated for 24, 48 and 72 hours. Subsequently, the cells were harvested by trypsinization and were resuspended in phosphate-buffered saline (PBS). The cell suspension was then mixed with an equal volume of 0.4% trypan blue solution prepared in PBS. The number of live cells (unstained) over the total number of cells was calculated as the percentage of viability (15).

### MTT assay

U87MG cells were cultured in a 96-well plates at a density of 1.5×10^4^ cells per well and were allowed to attachovernight. Then media containing different concentrationsof the extract were added to separate wells. After 24, 48 and 72 hours of treatment at 37°C and 5% CO_2_, the media were removed and 30 µL of MTT solution (5 mg/mL) was addedto each well, then incubated for 4 additional hours. Then 100 µL of dimethyl sulfoxide (DMSO) was added to dissolvethe formazan crystals produced by living cells at room temperature for 10 minutes with gentle shaking. The opticaldensity (OD) of resulting solutions was measured using anELISA reader at 570 nm with a reference wavelength of 630 nm. The percentage of cell viability was calculated according to the following formula (16):

Cell viability (%)=[OD_570, 630_ (sample)/OD_570, 630_ (control)]×100

The half maximal inhibitory concentration (IC_50_) values of *T. pratense* extract were obtained by nonlinear regression using GraphPad Prism 5 (GraphPad Software Inc, San Diego, USA). 

### Lactate dehydrogenase assay 

U87MG cells were seeded in 24-well plates and incubated overnight. Culture media (500 µl) containing different concentrations of *T. pratense* extract were added to separate wells, and the plates were incubated for 24, 48 and 72 hours. Then, 100 µl of medium from each sample was transferred to another plates and lactate dehydrogenase (LDH) activity was measured using Cytotoxicity Detection Kit (Roche Chemical Co., Germany) according to the manufacturer’s procedures. Finally, the OD at 490 nm with a reference wavelength of 690 nm for each sample was measured (17).

### Nitric oxide measurement

Griess reaction was used for evaluation of the effect of *T. pratense* extract on nitric oxide (NO) production by U87MG cells. After treatment with the different concentrations of the extract for 48 hours, the culture medium from each sample was collected. In order to remove the proteins, 100 µl of each sample was mixed with 6 mg of zinc sulfate and centrifuged at 10000 g for 10 minutes at 4ºC. Then 100 µl of each supernatant was mixed with 100 µl vanadium (III) chloride. Immediately Griess reagents [50 µl 2% sulfanilamide and 50 µl 0.1% N-(1-naphthyl) ethylenediamine dihydrochloride] were added and the samples were incubated for 30 minutes at 37ºC. The OD was measured by a microplate reader at 540 nm with a reference wavelength of 630 nm. The concentrations of NO were calculated from a sodium nitrite standard curve (18).

### Median effect analysis for TMZ and T. pratense extracts combination

The method proposed by Chou was used to determine andquantify the nature of TMZ and *T. pratense* extract interaction (synergistic, additive, or antagonistic) in a combination treatment. The combination of TMZ and *T. pratense* extract was prepared in constant concentration ratio (5.57:1) basedon their corresponding IC_50_ values in serial dilutions above and below the IC_50_ value of each agent, and then the MTTassay was performed. The combination index (CI) and dosereduction index (DRI) were calculated using CompuSynsoftware (ComboSyn, Inc., Paramus, NJ, USA). The CIvalues were interpreted as additive (CI=1), synergistic(CI<1) and antagonistic (CI>1). The DRI values representthe degree, to which the concentration of a compound can bereduced when used in combination with another compound, to maintain an equivalent effect. Finally, Fa is the fraction ofcell death ranging from 0 (no cell killing) to 1 (100% cell killing) (17).

### TUNEL staining 

Apoptosis was evaluated by labeling the 3´- hydroxyl termini in DNA fragments using an In Situ Cell Death Detection Kit, AP (Roche Diagnostics, Germany) according to the manufacturer’s instructions. After 48 hours of treatment with T. pratense extract in a 96 well plate, the cells were fixed with a freshly prepared paraformaldehyde solution (4% in PBS, pH=7.4) for 20 minutes at room temperature. Then the cells were rinsed with PBS and permeabilized with a 0.1% Triton X-100 solution in 0.1% sodium citrate for 5 minutes on ice (4°C). The cells were rinsed twice with PBS, and 50 µL of the TUNEL reaction mixture (label and enzyme solution) was added to each well, followed by incubation in a humidified chamber for 1 hour at 37°C. For differential staining of the cells a PI staining solution was used. The plate was incubated for 5 minutes at room temperature. The cells were then rinsed three times with PBS and analyzed under a fluorescentmicroscope (Nikon Corporation, Japan). All the mentionedstages are performed in the dark. The apoptotic index of the cells was calculated as follows (14):

Apoptotic index (%)=(number of apoptotic cells/total number of cells)×100 

### Acridin orange/ethydium bromide double staining

For observation of the intact, apoptotic and necrotic cellsunder the fluorescent microscope, AO/EB double stainingwas performed. AO passes through the plasma membrane ofcells and emits a green fluorescent light. EB only passes fromthe plasma membrane of cells when cytoplasmic membraneintegrity is lost, and emits a red fluorescent light. EB emissiondominates over AO. Therefore, live cells show uniform green nuclei, early apoptotic cells have yellow nuclei withfragmented chromatin, late apoptotic cells have fragmentedchromatin and orange nuclei,; and necrotic cells have solidorange nuclei (19). U87MG cells were cultured in 24-wellplates and treated with *T. pratense* extract. After 48 hours, cells were stained with mixture of AO/EB dye containing100 µg/ml of AO and 100 µg/ml of EB in PBS and observed under a fluorescent microscope (4). 

### Detection of acidic vesicular organelles

Autophagy induction was investigated by detection of acidicvesicular organelles (AVOs), which consist predominantlyof autophagosomes and autolysosomes. U87MG cells weregrown in the absence or presence of T. pratense extract for 48 hours in 24 well plates. Then the cells were stained with1 µg/ml AO (in PBS) for 20 minutes and were observedunder a fluorescent microscope. The percentage of the cellsgoing through autophagy was calculated using the following formula (14):

% of autophagic cells=(the number of cells with AVOs/ the total number of stained cells)×100

### Real-time polymerase chain rection 

The effects of various concentrations of *T. pratense* extract on *P53, CASPASE 3, BAX, BCL-2, LC3, ATG-7* and *BECLIN-1* mRNA expression were analyzed by real-timepolymerase chain reaction (PCR). Total RNA from GBMcells, treated with *T. pratense* extract for 48 hours, was prepared by total RNA isolation kit (DENAzist, Iran) and the quantity and quality of the extracted RNA were tested by Nano drop and gel electrophoresis. The complementary DNA (cDNA) synthesis was carried out using cDNA synthesis kit (Vivantis Technologies, Selangor DE., Malaysia). Real-time PCR was performed using SYBR Premix Ex Taq technology (Takara Bio Inc., Japan) on the Applied Biosystems StepOne Real Time PCR System (Life Technologies, USA). Glyceraldehyde 3-phosphate dehydrogenase (*GAPDH*) wasserved as an internal control and the fold change in relativeexpression of each target mRNA was calculated on the basisof comparative Ct (2^-ΔΔct^) method. Thermal cycler conditionswere 15 minutes at 50°C for cDNA synthesis, 10 minutes at95°C followed by 40 cycles of 15 seconds at 95°C to denaturethe DNA, and 45 seconds at 60°C to anneal and extend the template. The primer sequences were as follows:

CASPASE 3 F: 5´-CAAACTTTTTCAGAGGGGATCG-3´
  R: 5´-GCATACTGTTTCAGCATGGCAC-3´
P53 F: 5´-TAACAGTTCCTGCATGGGCGGC-3´
  R: 5´-AGGACAGGCACAAACACGCACC-3´
BCL-2 F: 5´-TTGTGGCCTTCTTTGAGTTCGGTG-3´
  R: 5´-GGTGCCGGTTCAGGTACTCAGTCA-3´
BAX F: 5´-CCTGTGCACCAAGGTGCCGGAACT-3´
  R: 5´-CCACCCTGGTCTTGGATCCAGCCC-3´
BECLIN-1 F: 5´-GCCGAAGACTGAAGGTCA-3´
  R: 5´-GTCTGGGCATAACGCATC-3´
LC3 F: 5´-GATGTCCGACTTATTCGAGAGC-3´
  R: 5´-TTGAGCTGTAAGCGCCTTCTA-3´
ATG-7 F: 5´-ATTGCTGCATCAAGAAACCC-3´
  R: 5´-GATGGAGAGCTCCTCAGCA-3´
GAPDH F: 5´-TCCCTGAGCTGAACGGGAAG-3´
  R: 5´-GGAGGAGTGGGTGTCGCTGT-3´.

### Statistical analysis 

All data are presented as mean ± SD of three independentexperiments. Statistical evaluation was done using one-wayanalysis of variance (ANOVA) with SPSS version 16.0(SPSS Inc., Chicago, IL, USA) software, and differences were considered to be statistically significant when P<0.05.

## Results

### Cell viability

**Fig.1:**
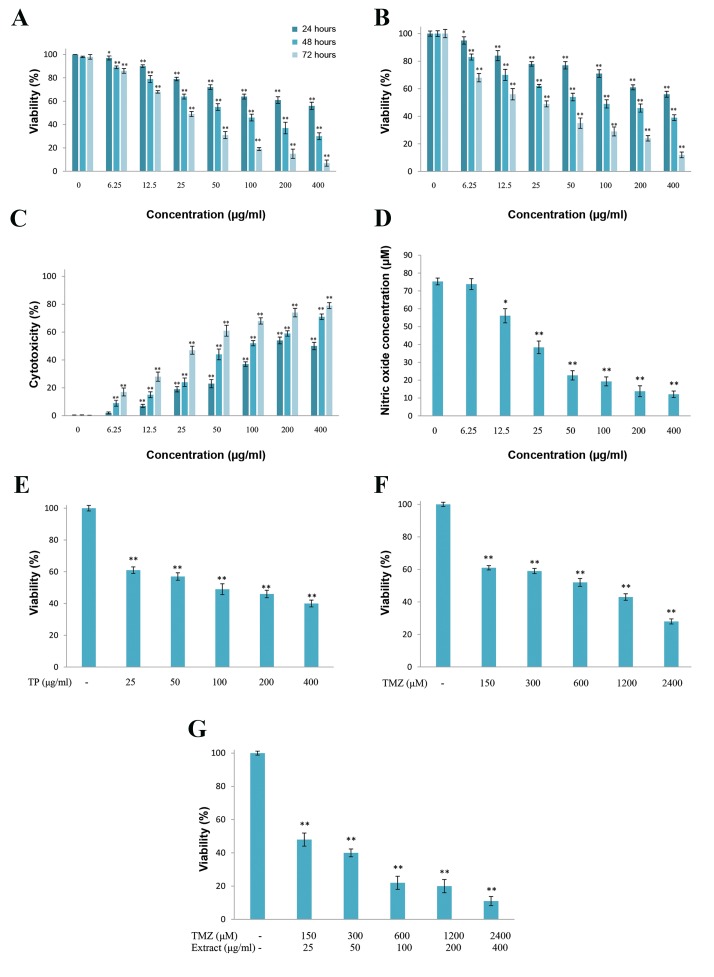
The effects of T. pratense extract on U87MG cells. Cell proliferation was determined using A. The MTT assay, B. Trypan blue staining, C. Lactate dehydrogenase (LDH) release from U87MG cells was measured colorimetric, D. Nitric oxide (NO) production was evaluated by Griess reaction, E. The effect of T. pratense extract, F. TMZ, and G. their combination on viability of U87MG cells after 48 hours of treatment were evaluated by MTT assay. The data are expressed as the percentage of control cells as the means ± SD. *; P<0.05 and **; P<0.01 compared with control.

The results of trypan blue staining and MTT assay after24, 48, and 72 hours showed a significant difference amongthe groups treated with T. pratense extract (6.25, 12.5, 25,50, 100, 200 and 400 µg/ml) compared to the control group(P<0.05). Increasing the dose significantly decreased cellviability (Fig .1A, B, P<0.05). So T. pratense extract reduced U87MG cell viability in dose- and time-dependent manner. The IC_50_ values for 24-, 48- and 72-hour treatments were 398.37, 109.19 and 21.06 µg/ml, respectively. 

### Cytotoxicity assay

Measurement of LDH activity in cell culture medium revealed that *T. pratense* extract significantly increased LDH release in dose- and time-dependent manners (Fig .1C, P<0.05). Therefore, cell death mediated by *T. pratense* extract is accompanied by plasma membrane damage.

### Nitric oxide levels 

The effects of different concentrations of *T. pratense* extract on U87MG cells after 48 hours of treatment showed a dose-dependent decrease in NO production. The difference compared to the control group was significant with the 12.5 µg/ml (P=0.02), 25 µg/ml (P=0.00), 50 µg/ ml (P=0.00), 100 µg/ml (P=0.00), 200 µg/ml (P=0.00) and 400 µg/ml (P=0.00) doses (Fig .1D).

### The effect of *T. pratense* extract treatment on temozolomide cytotoxicity 

Cancer cells were treated with a combination of TMZ and *T. pratense* extract for 48 hours (Fig .1E, F). Cell viability reduction by TMZ and *T. pratense* extract combination was greater than either TMZ or *T. pratense* extract alone (Fig .1G). In addition, CI and DRI values were calculated and listed in Table 1. The results showed that the CI values obtained in all tests were <1, indicating a synergistic effect. The DRI values for TMZ were >1 indicating a dose reduction for a given therapeutic effect. 

### TUNEL staining

**Fig.2:**
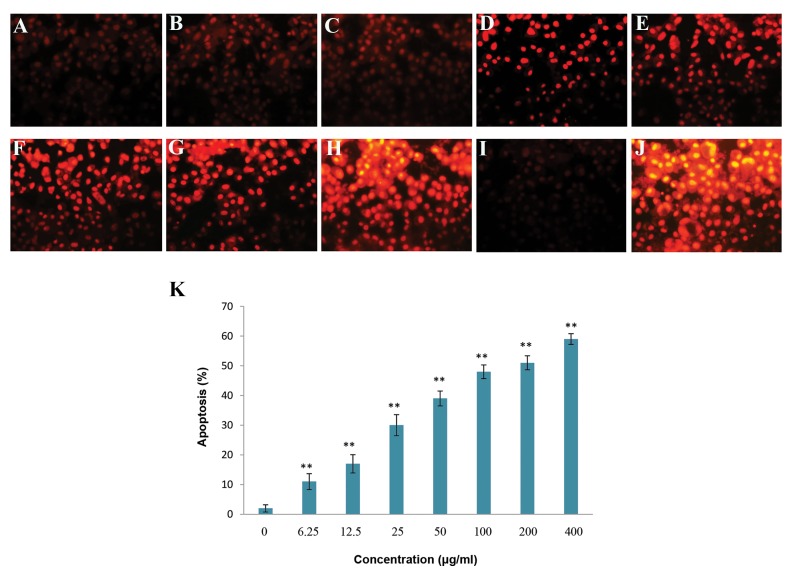
Apoptosis induction potential of T. pratense extract in U87MG cells was evaluated using TUNEL staining. A. Control cells, B. In the presence of 6.25 µg/ml, C. 12.5 µg/ml, D. 25 µg/ml, E. 50 µg/ml, F. 100 µg/ml, G. 200 µg/ml, H. 400 µg/ml of T. pratense extract, I. Positive control, J. Negative control, K. Columns mean percentage of apoptotic cells from three independent experiments. Negative control, positive control and control cells were treated with label solution without enzyme solution, DNase and serum free medium, respectively. The data are expressed as the percentage of the control cells as the means ± SD. *; P<0.05 and **; P< 0.01 compared with control.

The apoptosis index of U87MG cells treated with various concentrations of *T. pratense* extract for 48 hours showed that *T. pratense* increased apoptosis significantly in a dose-dependent manner (P<0.05). Apoptotic cell death was quantified and presented as percentage (Fig .2).

### Acridin orange/ethydium bromide staining

**Fig.3:**
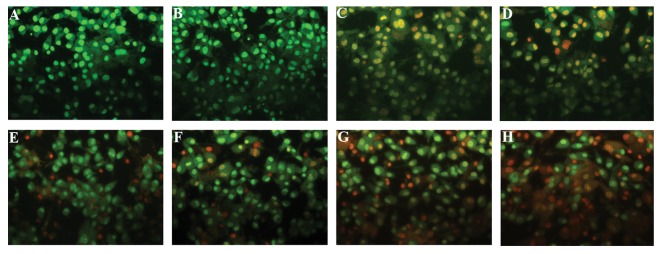
U87MG cells were stained with AO/EB and observed under fluorescent microscope. A. Control group, B. In the presence of 6.25, C. 12.5, D. 25, E. 50, F. 100, G. 200, and H. 400 µg/ml of T. pratense extract.

Morphological changes in apoptotic cells including cell shrinkage, chromatin condensation and nuclear fragmentation were detected using fluorescent dyes. Live cells with normal morphology were abundant in the control group, whereas early apoptotic cells were in cultures treated with 6.25 and 12.5 µg/ml (Fig .3). Both early and late apoptotic cells were observed in cultures treated with 25, 50, 100 and 200 µg/ml, and in the 400 µg/ml group most of the cells were in the late stage. Therefore, apoptosis increased in U87MG cells treated with T. pratense extract in a dose-dependent manner.

**Table 1:** Combination index (CI) and dose reduction index (DRI) values for temozolomide (TMZ) and Trifolium pretense extract combination


Temozolomide (μM)	Extract (μg/ml)	Fa	CI value	DRI Temozolomide	DRI extract

150	25	0.52	0.42	4.92	4.49
300	50	0.61	0.46	5.62	3.45
600	100	0.78	0.27	17.65	4.50
1200	200	0.81	0.43	13.35	2.79
2400	400	0.90	0.30	37.91	3.55


Fa; Fraction affected.

### Acidic vesicular organelles detection 

**Fig.4:**
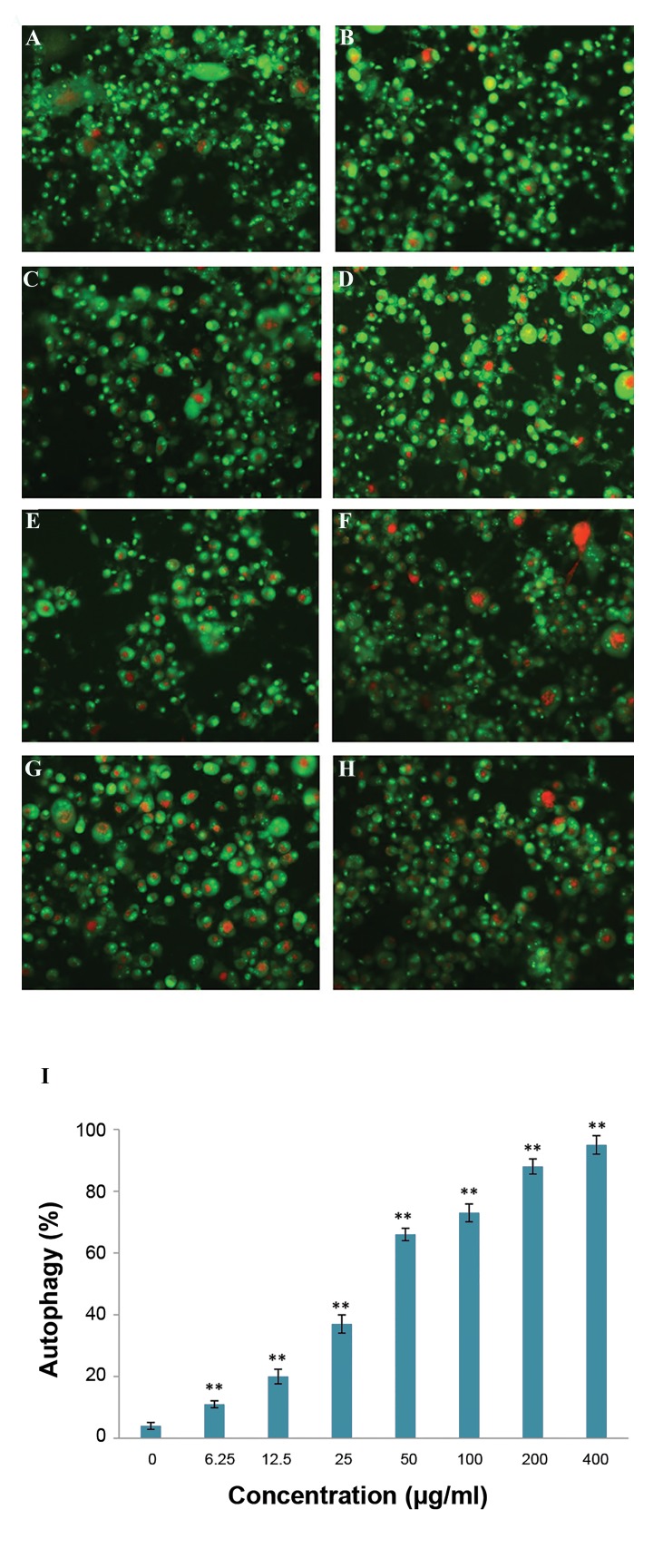
The effect of T. pratense extract on autophagy was investigated by AO staining in GBM cells. A. Control cells, B. In the presence of 6.25, C. 12.5, D. 25, E. 50, F. 100, G. 200, H. 400 µg/ml of *T. pratense* extract, and I. Columns mean percentage of autophagic cells from three independent experiments. Red dots indicate autophagic vesicles. The data are expressed as the percentage of the control cells as the means ± SD. *; P<0.05 and **; P<0.01 compared with control.

pratense increased autophagy significantly in a dose- The percentage of autophagy in U87MG cells treated dependent manner (P<0.05). Autophagy cell death were with *T. pratense* extract for 48 hours showed that *T. quantified* and presented as percentage (Fig .4). 

### Real-time polymerase chain reaction

**Fig.5:**
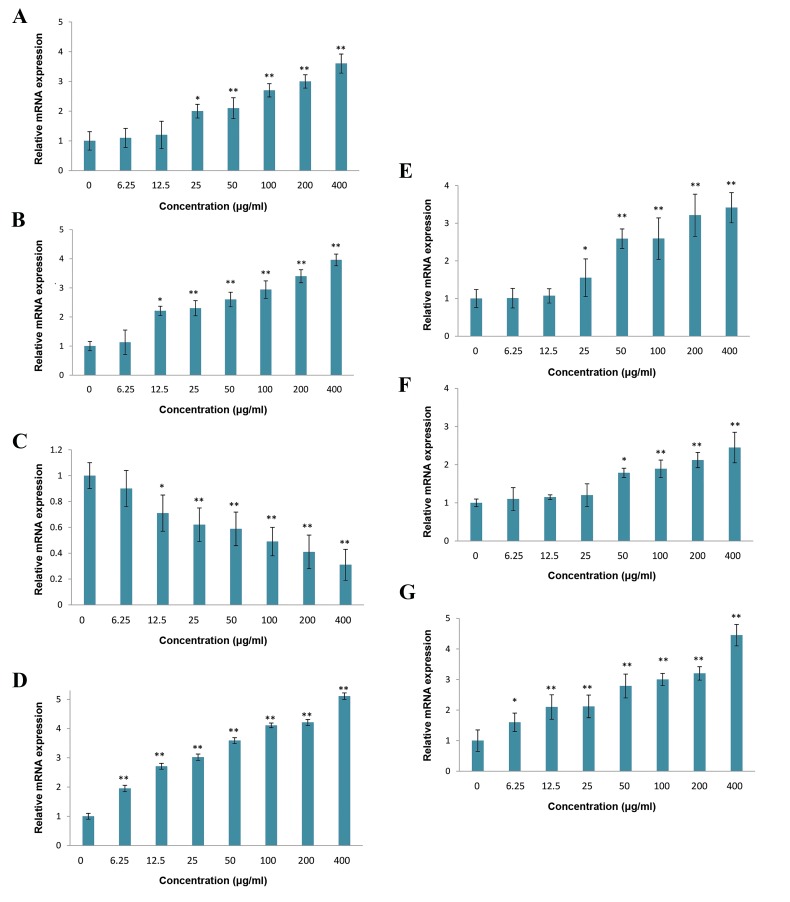
Expression levels of some apoptotic and autophagic factors in U87MG cells after treatment with different concentration of T. pratense extract for 48 hours was evaluated by real time PCR. A. P53 (tumorsuppressor), B. BAX (pro-apoptotic), C. BCL-2 (anti-apoptotic), D. CASPASE 3 (required enzyme for execution of apoptosis), E. BECLIN-1 (key positive autophagic regulator), F. ATG-7 (essential autophagy gene), and G. LC3 (essential for autophagosome formation). The data are expressed in terms of percent of control cells as the means ± SD. *; P<0.05 and **; P<0.01 compared with control.

Expression of some apoptosis- and autophagy-related genes was evaluated using real time PCR. *P53* was upregulated in cells that were treated with *T. pratense* extract (Fig .5A). The results of real time PCR also suggested a downregulation of *BCL-2* and upregulation of *BAX* mRNA expression after 48 hours exposure to *T. pratense* extract (Fig .5B, C). Exposure of U87MG cells to *T. pratense* extract led to increased mRNA expression of *CASPASE 3* gene (Fig .5D). Also, the extract increased the LC 3, *BECLIN-1* and *ATG-7* mRNA levels (Fig .5E-G). Thus, *T. pratense* extract induced apoptotic and autophagy in U87MG cells at the transcriptional level.

## Discussion

The aim of the current study was to evaluate the effects of *T. pratense* extract on GBM cells. First, the potentials of seven different concentrations of this extract to promote cell death were tested. Our results showed that *T. pratense* hydroalcoholic extract decreased cell viability in a time- and dose-dependent manner. We also investigated whether *T. pratense* extract could have a therapeutically beneficial effect when administered in combination with TMZ (conventional chemotherapy agent for GBM). Interestingly, the results of this study showed that *T. pratense* extract increased the cytotoxicity of TMZ and a combination of the extract with TMZ demonstrated synergistic effects on U87MG cell line proliferation with CI values between 0.27 and 0.46. The mean CI of all tests in the present study was 0.37. In other words, TMZ and *T. pratense* extract acted synergistically to reduce the viability of GBM cells. This combination also resulted in a noticeable dose reduction for TMZ and reduced its IC_50_ to about 4.27 fold smaller. TMZ, like many other chemotherapeutic drugs, produces different types of general side effects such as moderate to severe lymphopenia or abnormally low levels of white blood cells. Therefore, a dose reduction of TMZ for therapy is clinically very important.

Further, this research showed that *T. pratense* extract induced both apoptosis and autophagy in U87MG cells. Apoptosis is a programmed cell death and characterized by morphological and biochemical. This kind of cell death acts as a homeostatic mechanism. Cells with defective or inefficient apoptosis pathway are enabling to survive even under oxidative stress or hypoxia. Induction of apoptosis can be an appropriate strategy, by which anti-cancer agents destroy tumor cells (20). Autophagy is a catabolic process that is essential for development, differentiation, survival and homeostasis, and allows cells to degrade and recycle of cellular components via lysosomal enzyme. A number of studies have indicated that anti-cancer agents induce autophagy in human cancer cells (21).

In the present study, our findings showed that the number of AVO-containing cells was increased in a dose- dependent manner, indicating the induction of autophagy. Also, the number of TUNEL-positive cells was increased in a dose-dependent manner, indicating the induction of apoptosis. At a molecular level, the mRNA expression of some autophagy- and apoptosis-related genes were significantly changed by *T. pratense* extract treatment in GBM cells. T. pratense extract treatment increased the expression of *P53, BAX, CASPASE 3, LC3, BECLIN-1* and *ATG-7*. The expression level of *BCL-2* was reduced by *T. pratense* extract treatment. These results were in agreement with the findings of AVO and TUNEL staining.

*P53* is a tumor suppressor protein, involved in both apoptosis and autophagy cell death. P53 increases the expression of *BAX* and reduces the expression of *BCL-2* genes. The ratio of pro-apoptotic *BAX* to anti-apoptotic *BCL-2* protein controls the intrinsic pathway of apoptosis (22). Increased *BAX /BCL-2* ratio up-regulates *CASPASE* 3 expression and induces apoptosis cell death (23). P53 induces autophagy through TOR inhibition and also through transcriptional activation of DRAM (24). P53 can also induce autophagy by regulation of *LC3. LC3* is an essential protein in autophagy pathway (25). *BECLIN-1* is the other essential protein in autophagy pathway that has an impotent roll in autophagosome formation (26). Also *ATG-7* is another autophagy-promoting gene involving in regulation of autophagy (27). 

NO has been reported to be involved in many physiological and pathological processes in the brain and plays a dual and critical role in glioma biology (28). This research indicated that *T. pratense* extract significantly reduced NO production by GBM cells. NO is a signaling molecule with complex regulatory effects on both physiological and pathological conditions (29). So, modulation of NO production in cancer cells can potentially be a good strategy to achieve anti-glioma effects. Previous studies have shown that cell proliferation, vascularization, invasion, chemo-and radiotherapy sensitivity and immune reactivity in glioma tumors can be affected by NO concentration (30).

Also, NO is a bifunctional regulator of apoptosis. Proapoptotic and anti-apoptotic functions of NO have been reported in various *in vivo* and *in vitro* experimental models (31). Studies have shown that NO can be an important endogenous inhibitor of apoptosis (32). Among the most important anti-apoptotic activities of NO are induction of cytoprotective stress proteins, cGMP-dependent inhibition of apoptotic signal transduction, suppression of *CASPASE* activity and inhibition of cytochrome c release (31). NO also inhibits *CASPASE* activation and apoptotic morphology in neurons. However, to this point, there has not been any studies on the role of NO in glial cells apoptosis (33). Our data indicated that *T. pratense* extract reduced NO production and may remove the antiapoptotic effect of NO in U87MG cells.

As previously stated, *Trifolium pratense L.* extract was composed of isoflavones, flavonoids, pterocarpans, coumarins and tyramine. Its main isoflavones are biohanin A, formononetin, daizdein, genistein, pratensein, prunetin, pseudobaptigenin, calycosin, methylorobol, afrormosin, texasin, irilin B and irilone (12). Dietary flavonoids are the most abundant polyphenols in plant sources. Several plant-derived flavonoids (silymarin, genistein, quercetin, daidzein, luteolin, kaempferol, apigenin, and epigallocatechin 3-gallate) have been reported to have an anti-proliferative effect on various cancers such as prostate, colorectal, breast, thyroid, lung, and ovarian. Their anti-cancer effect is mediated by activation of apoptosis, cell cycle arrest, inhibition of metabolizing enzymes, reactive oxygen species formation, vascular endothelial growth factor and basic fibroblast growth factor. Also, some flavonoids have been reported to reduce cancer cells drug resistance (34).

Genistein and *daidzein*, two member of flavonoid family, have noticeable anti-proliferation effects against breast cancer, due to their structural similarity with estrogen. Anti-cancer effects of quercetin, another member of flavonoid family against colon cancer and glioma tumors, is mediated by activation of autophagy signaling pathway. Nowadays, a variety of these flavonoids are used in dietary supplements, but none of them have been approved for clinical use (34). From the pterocarpans family, indigocarpan has shown anti-proliferative activities in human cancer cell lines via induction of a *CASPASE* -dependent apoptosis pathway (35).

The anti-cancer activity of coumarins is mediated by various pathways including inhibition of kinase, cell cycle progression, angiogenesis, heat shock protein-90 (HSP90), telomerase, mitotic activity, carbonic anhydrase, monocarboxylate transporters, aromatase and sulfatase (36). Despite considerable progress in cancer therapy over the past decades, GBM is still associated with very poor prognosis, and few patients survive more than 3 years due to inherent chemo-resistance. Therefore, development of new treatment strategies is essential for the patients with GBM. Our data suggests for the first time that *T. pratense* extract enhances the anti-neoplastic effect of TMZ in GBM predominantly by augmentation of apoptosis and autophagy of cancer cells.

## Conclusion

Trifolium Pratens is potentially beneficial for further development of new chemotherapeutic agents. The present data open a new possible approach in the cure of GBM. Future studies are necessary to seek if a combined treatment with *T. pratense* extract and TMZ provide better results in *in vivo* models.
